# Modulating and opposite actions of two aqueous extracts prepared from *Cinnamomum cassia* L. bark and *Quercus ilex* L. on the gastrointestinal tract in rats

**DOI:** 10.1039/c9ra02429h

**Published:** 2019-07-12

**Authors:** Hichem Sebai, Kaïs Rtibi, Slimen Selmi, Mourad Jridi, Rafik Balti, Lamjed Marzouki

**Affiliations:** Laboratory of Functional Physiology and Valorization of Bio-resources, Higher Institute of Biotechnology of Beja, University of Jendouba B. P. 382 9000 Beja Tunisia rtibikais@yahoo.fr +216 72 590 566 +216 97 479 135; Laboratory of Enzymatic Engineering and Microbiology, National School of Engineers of Sfax, University of Sfax B. P. 1173 3038 Sfax Tunisia

## Abstract

Cinnamon bark and evergreen oak acorns, natural sources of functional ingredients, are effective for those suffering from diarrhea, constipation and irritable bowel syndrome. This study aimed to evaluate the dissimilar phytochemical composition and the opposite potential actions of *Cinnamomum cassia* bark (ACCE) and *Quercus ilex* aqueous extracts (GIAE) on gastrointestinal (GI)-physiological activities and disruptions. An HPLC-PDA/ESI-MS assay was used to identify the distinctive qualitative and quantitative profiles of phenolic compounds. The GI-physiological action of both extracts on gastric emptying (GE) and gastrointestinal transit time (GIT) were evaluated using the phenol-red colorimetric method and a test meal containing charcoal/gum arabic in water. Loperamide (LOP)-induced colonic constipation and delayed emptying of the stomach were used to explore the reverse effects of ACCE/GIAE on GI disorders. HPLC-PDA/ESI-MS showed that the main phenolic compounds detected in ACCE are *trans*-cinnamic acid, quinic acid, protocatechuic acid and rosmarinic acid, while gallic acid, quinic acid and protocatechuic acid are the major chemical constituents found in GIAE. GIAE at two doses (150 and 300 mg kg^−1^) exerted a reduction of GE (66.40% and 60.87%, respectively) compared to a control group (70.25%). However, ACCE at the same concentrations induced contradictory actions on GE/GIT in comparison to GIAE and antagonistic synthetic pharmacological drugs in rats. The protective effect of CCAE against constipation induced by LOP in rats was accompanied by a strong antioxidant property related to moderation of intracellular-mediator disorders. An absence of toxic actions was revealed in the case of the hematological profile and biochemical parameters. Hence, in-depth investigations of these nutrients of both extracts may help future researchers to derive the underlying mechanisms and potential molecular targets for the development of physiologically functional foods and future therapies.

## Abstract 2

Cinnamon bark and evergreen oak acorns, natural sources of functional ingredients, are effective for those suffering from diarrhea, constipation and irritable bowel syndrome.

## Graphical abstract

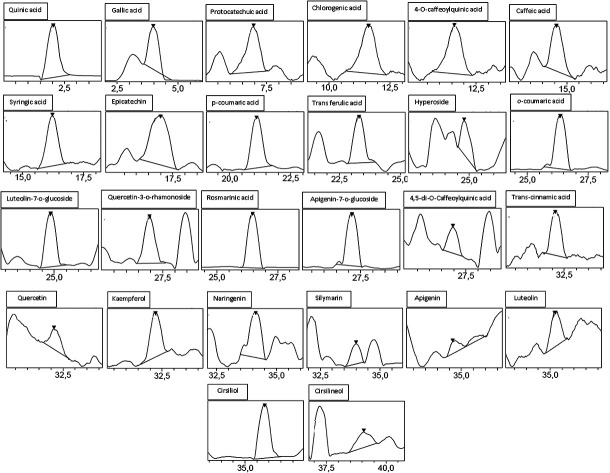

## 1. Introduction

Plants have diverse defense strategies in the fight against a huge variety of damaging biotic circumstances.^1^ Polyphenol compounds are largely implicated in the protection system against ultraviolet radiation and pathogens. In foods, polyphenols participate in many functions, such as color, flavor, odor, bitterness, and astringency, and exhibit antioxidant properties. Indeed, several current biomedical studies related to these compounds and the great antioxidant capacity of a number of herbal preparations and foods show that polyphenols, such as phenolic acids, flavonoids, tannins, and proanthocyanidins, can provide nutritional protection against various chronic disorders in humans and animals, including diarrhea, constipation, cardiovascular diseases, cancers, diabetes, and neurodegenerative diseases.^2^

The powdered bark of *Cinnamomum cassia* has long been consumed as a spice for flavoring foods as well as in traditional remedies. The *C. cassia* tree belonging to the Lauraceae family, originates from southern China, Bangladesh, Uganda, India, and Vietnam.^3^ It is used to protect against or treat many diseases, as well as to maintain health and well-being. Indeed, cinnamon polyphenols may have additional benefits for human health. Recently, studies have shown that *C. cassia* has diverse bioactivities, including antioxidant,^4^ antimicrobial,^5^ anticancer,^6^ anti-inflammatory,^7^ as well as pharmacological properties in the treatment of type II diabetes. In the digestive tract, cinnamon has an anti-tumor activity in colon cancer and has been reported to decrease the risk of a gastric ulcer as well as protecting the gastrointestinal system from free radical damage.^8,9^

*Quercus ilex* L. (the evergreen oak) belongs to the family of Fagaceae, and is distributed throughout France, Italy, Croatia, Greece and North Africa, including Morocco, Algeria and especially in northern Tunisia. Oak kernels are traditionally used throughout Tunisia, and other countries, for treating diarrhea and diabetes. These fruits have long been employed as a source of tannin, oil, and especially food because of their content of carbohydrates, amino acids, proteins, lipids, and various sterols.^10,11^

The gastro-intestinal system is essentially a long tube running right through the body, with specialised sections that are capable of digesting food put in at the top end and extracting any useful components from it *via* an absorption process, then expelling the waste products. The whole system is under the control of the enteric nervous system and hormones.

Functional gastrointestinal disorders (FGID), such as irritable bowel syndrome (IBS) and functional dyspepsia, delayed gastric emptying (DGE), constipation and diarrhea are distinguished by aggravating gastrointestinal ailments in the absence of an underlying organic cause that can readily explain the symptoms.^12–14^ Currently, several mechanisms have been suggested to be involved in the pathophysiology of FGIDs. These include perturbations of the visceral sensory function, dietary change, genetic factors, infections and interruptions in the intestinal microbiota, low-grade mucosal inflammation, immune activation, and modified intestinal permeability.^15^

The aim of this study is to compare the chemical composition and potential GI-physiological actions of both extracts prepared from cinnamon bark and evergreen oak acorn as well as the possible underlying mechanisms of the actions.

## 2. Materials and methods

### 2.1. Drugs and reagents

Gallic acid (PubChem CID: 370), quercetin (PubChem CID: 5280343), tannic acid (PubChem CID: 16129778), methanol (PubChem CID: 887), sodium chloride (PubChem CID: 5234), diethyl ether (PubChem CID: 3283), formic acid (PubChem CID: 284), sodium pentobarbital (PubChem CID: 23676152), clonidine (PubChem CID: 2803), yohimbine hydrochloride (PubChem CID: 6169), loperamide (PubChem CID: 3955), haematoxylin (PubChem CID: 442514), eosine (PubChem CID: 11048), charcoal meal (PubChem CID: 297), sodium hydroxide (PubChem CID: 14798) and formaldehyde (PubChem CID: 712) were obtained from the Sigma Chemical Co. (Sigma-Aldrich GmbH, Steinheim, Germany).

### 2.2. Preparation of cinnamon bark and oak acorn extracts

Cinnamon bark and oak acorns were obtained from the local market (Distributor, Tunisia) and identified by the botanic coordinator, Institute of Biotechnology of Beja, University of Jendouba. Plant materials were dried at 40 °C with air circulation and ground into a fine powder using a laboratory blender. The active materials were extracted from the powder into an aqueous phosphate buffer solution 0.02 M, pH 7 (1/5; w/v) overnight under magnetic agitation for 24 h and filtered through a colander (0.5 mm mesh size). The obtained extracts were dehydrated at the same temperature under vacuum and finally freeze dried. The yield of the extracts was calculated in grams and converted into a percentage. In the case of GIAE, the percentage yield of the evergreen oak acorn extract was recorded as approximately 10%. However, the yield of extraction of ACCE was 5%.

### 2.3. Identification of distinctive phenolic compounds by liquid chromatography-high resolution electrospray ionization mass spectrometry (LC-HRESIMS) assay

100 mg of each extract (GIAE and ACCE) were dissolved in 100 mL of 10% methanol and filtered and then 1 mL was transferred into LC-MS vials. An opposite-phase column (Pursuit XRs ULTRA 2.8, C18, 100 × 2 mm, Agilent Technologies, UK) was used to carry out HPLC investigations. 20 mL of the prepared samples were injected at a column temperature set at 30 °C. Mobile phases consisted of 0.1% formic acid in water (A) and 0.1% formic acid in methanol (B). A gradient program was used for isolation at a flow rate of 1 mL min^−1^. Mobile phases consisted of an initial composition of 100% solvent A, with a gradient of 100% solvent B over 20 minutes, held at 100% solvent B for 5 min and 100% solvent A for 25 min. The drying gas flow rate was 1 mL min^−1^ at 320 °C. MS was operated in the positive ion mode in a mass range of 100–2000 *m*/*z*. High resolution mass spectral data were obtained on a Thermo Instruments ESI-MS system (LTQ XL/LTQ Orbitrap Discovery, UK) connected to a Thermo Instruments HPLC system (Accela PDA Detector, Accela PDA Autosampler and Accela Pump).^16^

### 2.4. Animals and ethics statement

This study was performed in strict accordance with the NIH guidelines for the care and use of laboratory animals and was approved by the Institutional Animal Care and Use Committee of the National Institute of Health.^17^

Male Wistar rats (weighing 180–220 g) and male mice (weighing 20–30 g) were obtained from the Central Society of Pharmaceutical Industries of Tunisia (SIPHAT, Ben-Arous, Tunisia). The animals were separated into different groups and acclimatized for 15 days with a standard pellet diet (standard pellet diet Badr-Utique-TN) and water *ad libitum* (22 ± 2 °C; 12 h dark/light cycle).

### 2.5. Acute toxicity test

An acute toxicity study was carried out according to precedent.^18^ Both extracts were administered orally to different groups of mice in increasing doses ranging from 50 to 3000 mg kg^−1^. A control group received 10 mL kg^−1^ of NaCl (0.9%) *via* the same routes. The animals were placed under observation for 24 hours to monitor their behavior and mortality. Based on results of LD_50_, 1/10th and 1/20th of the LD_50_ were taken as therapeutic doses.^18^

### 2.6. Sub-acute toxicity study

The rats were divided into five groups (*n* = 6). Group I received 10 mL kg^−1^ of NaCl (0.9%) orally and served as a control group, whereas the rats in groups II, III, IV and V were treated with ACCE or GIAE at the doses of 300 mg kg^−1^ and 700 mg kg^−1^ body weight continuously up to 21 days. At the end of the experiment, blood samples were collected from the rats after an overnight fast (but with drinking water allowed) by retro-orbital bleeding into heparinized and non-heparinized tubes for hematological and biochemical investigations, respectively.^19^

### 2.7. Hematological and biochemical parameters

The heparinised blood was used for the analysis of hematological parameters, such as hemoglobin, red blood cell count, white blood cell count, and platelet count, using an automated hematology analyzer. However, the separated serum from non-heparinized blood was used to estimate biochemical parameters, including triglyceride, total cholesterol, creatinine, alanine aminotransferase (ALT), and aspartate aminotransferase (AST) levels using commercial kits purchased from Biomaghreb, Tunisia.

### 2.8. Gastric emptying and extract/drug action analysis

The phenol-red technique was used to investigate the stomach-emptying activity in the vehicle-group and after extract/drug administration in rats. For this reason, the animals used were separated into six lots with six–eight in each and treated 1 h before a test meal (50 mg of phenol red in 100 mL of aqueous methyl cellulose) as follows:

Group 1 served as a vehicle-group (VC) and received 1 mL of physiological solution (NaCl, 0.9%, p.o.).

Groups 2 and 3 were administered by the gavage method with two doses of GIAE (150 and 300 mg kg^−1^, b.w. p.o.).

Groups 4 and 5 were orally pre-treated with two doses of ACCE (150 and 300 mg kg^−1^, b.w. p.o.).

Group 6 was pre-treated with the standard drug (LOP, 5 mg kg^−1^, b.w. p.o.).

1 h later, the rats were anesthetized and euthanized. The gastric regions and their contents were combined with 100 mL of NaOH (0.1 N). The obtained supernatants were mixed with 4 mL of NaOH (0.5 N) and the absorbance of the samples was read at 560 nm. Phenol-red collected from euthanized animals directly after administration of the test meal was used as a standard (0% emptying).^20^

The gastric-emptying percentage was determined according to the following formula:Gastric emptying rate (%) = (1 − absorbance of treated/absorbance of standard) × 100.

### 2.9. Small bowel transit and extract/drug action investigation

Assessment of gastrointestinal propulsion was determined according to experimental method followed in our previous study using the charcoal meal test.^21^ In short, two hours after intragastric administration of separate extracts of both at doses of 150 mg kg^−1^, and 300 mg kg^−1^, the diverse groups of rats received a standard charcoal meal (10% charcoal in 5% gum arabic) by stomach administration. At 30 min after administration of the charcoal meal, the animals were anesthetized, a laparotomy was performed and the small bowels were excised from the gastroduodenal junction to the ileocecal junction. The distance traveled by the meal was measured and expressed as a percentage of the total length of the small intestine from both junctions. Yohimbine (an α-2 adrenergic-antagonist) and clonidine (an α-2 adrenergic-agonist) were used as antagonistic standard drugs at doses of 2 mg kg^−1^ and 1 mg kg^−1^, respectively. The peristaltic index and inhibition percentages were calculated using the following formulae:Peristaltic index = (distance travelled by charcoal meal/length of small intestine) × 100% of inhibition = mean distance travelled by the control − mean distance travelled by the test group/mean distance travelled by the control.

### 2.10. (LOP)-drug-caused constipation and extract influence assays

Constipation was caused in animals by subcutaneous injection of loperamide in 0.9% sodium chloride once a day for one week, according to previous reports.^22^ In this case, a total of 32 rats were randomly divided into 4 groups (*n* = 6 per group): vehicle or normal control-group (VC), model control group (MC) and 2 groups of rats which were treated with LOP in association with ACCE (150 and 300 mg kg^−1^, b.w. p.o.). VC: treated with normal saline (10 mL kg^−1^); MC: treated only with LOP (5 mg kg^−1^ of body weight); LOP + ACCE: treated with LOP (5 mg kg^−1^) and ACCE at both doses.

Disturbances in food intake, water intake, and body weight were measured daily at 09:00 am throughout the experimental period using an electronic balance. All evaluations were accomplished three times to ensure accuracy.

To examine the fecal parameters, animal feces were collected and counted during the experimental measurements. The wet weight of the feces was recorded immediately after collection, and the dry weight of feces was reported after they had been dried in a drying oven for 3 h.

After 7 days of treatment, the rats were fasted for 12 h but allowed free access to water. The colons were excised immediately and flushed with normal saline at 4 °C, and were then stored at −80 °C until they were assayed.

### 2.11. Disturbance of free radical production/antioxidant defense balance assessment

An increase in the production of reactive oxygen species has been estimated by oxidative modifications of cellular components, such as polyunsaturated fatty acids and proteins, leading to the generation of malondialdehyde (MDA) and protein-carbonyl groups. In this context, colonic mucosa MDA contents were estimated using the double heating method.^23^ Briefly, mucosal supernatants were combined with a BHT–TCA solution composed of 1% BHT (w/v) and 20% TCA (w/v). The mixture was centrifuged at 1000 × *g* for 5 min at 4 °C. The obtained supernatants were blended with 0.5 N HCl and 120 mM of TBA in 26 mM of Tris and then heated in a laboratory water bath at 80 °C for 10 min. After rapid cooling, an optical density absorbance reading was obtained with a UV-vis spectrophotometer (Beckman DU 640B) at 532 nm. MDA levels were calculated using an extinction coefficient for the MDA–TBA complex of 1.56 × 10^5^ M^−1^ cm^−1^. The creation of protein carbonyls in constipated rats caused by LOP-induced constipation was estimated according to the design of Levine *et al.* (1990)^24^ and the data were expressed as μmol carbonyl residues per mg proteins. Likewise, (–SH) group levels were studied using Ellman's method (1959)^25^ and the results were expressed as nmol of thiol groups per mg of proteins.

The involvement of the enzymatic system to reduce this oxidative damage was evaluated by the change in the activity of the principal defensive agents or antioxidant enzymes, such as superoxide dismutase, catalase and glutathione peroxidase. For this reason, (SOD)-activity was measured by the technique of reduction of the nicotinamide adenine dinucleotide (reduced) phenazinemetho-sulphate-nitroblue-tetrazolium reaction system attributed to Kakkar *et al.* (1984)^26^ and the data were expressed as units (U) of SOD activity per mg of proteins. (CAT)-activity was approximated by the method of Aebi (1974)^27^ and the results are expressed as nmol min^−1^ mg^−1^ proteins.

### 2.12. Moderation of intracellular mediators in examinations of constipated rats

To examine colonic-cell disruption, the H_2_O_2_ level was measured according to the method of Dingeon *et al.* (1975).^28^ The non-haem iron was studied using ferrozine, as described by Leardi *et al.* (1998).^29^ The calcium level was determined using a colorimetric method, according to Stern and Lewis (1975).^30^

### 2.13. Statistical evaluations

A one-way analysis of variance test was used to determine the significant differences between the different groups of all animals. Statistical analyses were calculated using StatView statistical software. The data are representative of six to eight distinct observations. Differences were stated as mean ± SEM and designated significant when the values of *p* were less than 0.05.

## 3. Results

### 3.1. HPLC-PDA/ESI-MS identification of phenolic compounds in both extracts

**Fig. 1:**
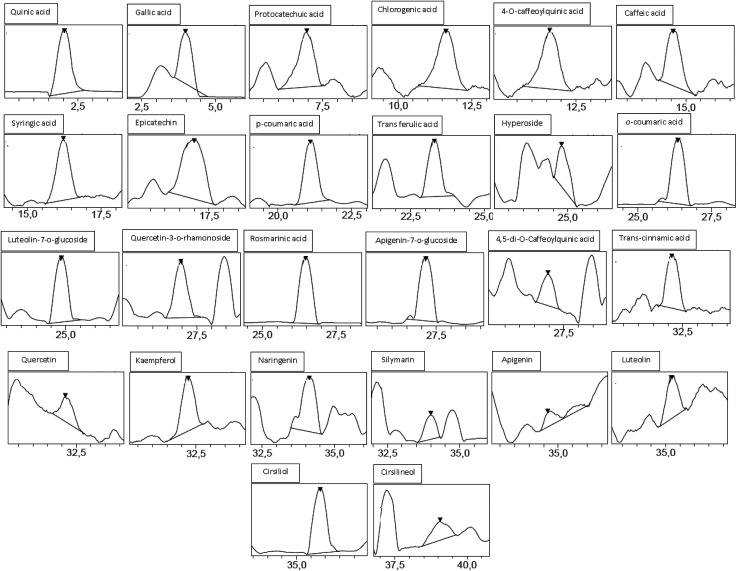
Chromatographic profile and characterization of phenolic compounds of ACCE.

**Fig. 2:**
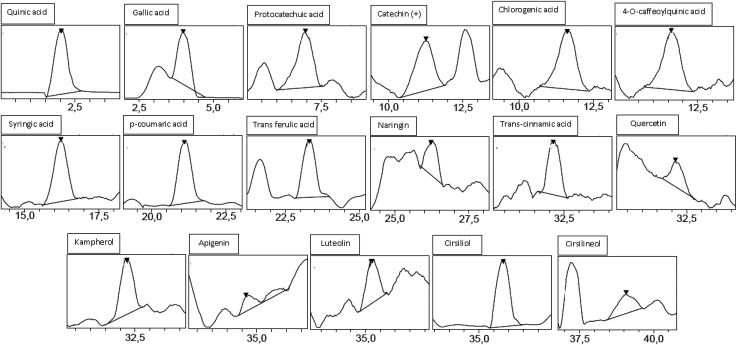
Chromatographic profile and characterization of phenolic compounds of GIAE.

Analysis by HPLC-PDA/ESI-MS revealed 26 phenolic compounds (Table 1) in ACCE: *trans*-cinnamic acid, quinic acid, protocatechuic acid, rosmarinic acid, epicatechin, *p*-coumaric acid, cirsiliol, syringic acid, gallic acid, *trans*-ferulic acid, 4-*O*-caffeoylquinic acid, chlorogenic acid, luteolin-7-*o*-glucoside, *o*-coumaric acid, caffeic acid, quercetrin (quercetin-3-*o*-rhamonosid), apigenin-7-*o*-glucoside, silymarin, hyperoside (quercetin-3-*o*-galactoside), luteolin, quercetin, 4,5-di-*O*-caffeoylquinic acid, apigenin, naringenin, kaempferol and cirsilineol. In contrast, only 17 phenolic compounds (Table 1) were detected in the other extract (GIAE) obtained from the oak acorns which contained gallic acid, quinic acid, protocatechuic acid, *p*-coumaric acid, syringic acid, naringin, cirsiliol, *trans*-ferulic acid, *trans*-cinnamic acid, catechin(+), 4-*O*-caffeoylquinic acid, chlorogenic acid, quercetin, cirsilineol, luteolin, kaempferol and apigenin. There are 15 compounds that are similar between the two extracts, but they occur in different amounts which explains why both extracts can exert different biological effects (Fig. 1 and 2).

**Table 1:** High-resolution liquid chromatography/electrospray ionization (LC-HRESIMS) identification of both extracts (ACCE and GIAE)

Name^a^	Molecular formula	PubChem CID	[M]^−^H *m*/*z*^b^	ACCE		GIAE	
				Retention time	Concentration (ppm)	Retention time	Concentration (ppm)
Quinic acid	C_7_H_12_O_6_	6508	191.00	2.011	191.462	2.037	307.485
Gallic acid	C_7_H_6_O_5_	370	169.00	3.986	4.198	3.989	358.708
Protocatechuic acid	C_7_H_6_O_4_	72	153.00	6.925	155.249	6.955	8.606
Catechin(+)	C_15_H_14_O_6_	73160	289.00	—	ND	11.260	0.469
Chlorogenic acid	C_16_H_18_O_9_	1794427	353.00	11.602	2.762	11.624	0.423
4-*O*-Caffeoylquinic acid	C_16_H_18_O_9_	5281780	353.00	11.602	2.821	11.624	0.442
Caffeic acid	C_9_H_8_O_4_	689043	179.00	14.581	1.587	—	ND
Syringic acid	C_9_H_10_O_5_	10742	197.00	16.206	6.281	16.216	2.727
Epicatechin	C_15_H_14_O_6_	72276	289.00	16.993	10.063	—	ND
*p*-Coumaric acid	C_9_H_8_O_3_	637542	163.00	21.017	7.184	21.108	7.120
*trans*-Ferulic acid	C_10_H_10_O_4_	445858	193.00	23.269	2.845	23.317	0.954
Hyperoside (quercetin-3-*o*-galactoside)	C_21_H_20_O_12_	5281643	463.00	24.792	0.438	—	ND
*o*-Coumaric acid	C_9_H_8_O_3_	637540	163.00	26.367	1.792	—	ND
Luteolin-7-*o*-glucoside	C_21_H_20_O_11_	5280637	447.00	24.853	1.913	—	ND
Quercetrin (quercetin-3-*o*-rhamonoside)	C_21_H_20_O_11_	15939939	447.00	26.958	0.734	—	ND
Rosmarinic acid	C_18_H_16_O_8_	5315615	359.00	26.473	86.058	—	ND
Naringin	C_27_H_32_O_14_	442428	579.00	—	ND	26.218	2.256
Apigenin-7-*o*-glucoside	C_21_H_20_O_10_	45933926	431.00	27.147	0.567	—	ND
4,5-Di-*O*-caffeoyquinic acid	C_25_H_24_O_12_	6474309	515.00	26.961	0.269	—	ND
*trans*-Cinnamic acid	C_9_H_8_O_2_	444539	147.00	32.158	1105.222	32.133	0.919
Quercetin	C_15_H_10_O_7_	5280343	301.00	32.140	0.292	32.124	0.063
Kaempferol	C_15_H_10_O_6_	5280863	285.00	32.197	0.156	32.228	0.023
Naringenin	C_15_H_12_O_5_	439246	271.00	34.130	0.196	—	ND
Silymarin	C_25_H_22_O_10_	31553	481.00	34.000	0.516	—	ND
Apigenin	C_15_H_10_O_5_	5280443	269.00	35.016	0.215	34.633	0.019
Luteolin	C_15_H_10_O_6_	5280445	285.00	34.718	0.386	35.222	0.027
Cirsiliol	C_17_H_14_O_7_	160237	329.00	35.809	6.451	35.820	1.559
Cirsilineol	C_18_H_16_O_7_	162464	343.00	39.093	0.040	39.073	0.033

a The compounds are suggested according to the dictionary of natural products and the characteristic fragmentation pattern.

b The formulae were deduced from the quasi molecular ion peak [M + H]^+^.

### 3.2. Acute/sub-acute toxicities of ACCE/GIAE

Oral administration of both extracts at different doses did not produce any clinical signs of toxicity or death in the mice. LD_50_ values of ACCE/GIAE were found to be greater than 3000 mg kg^−1^. A similar absence of toxic actions was revealed in the case of the hematological profile and biochemical parameters during the 3 weeks of treatment of animals with the extracts (Tables 2 and 3).

**Table 2:** Effects of ACCE and GIAE on hematological parameters in sub-acute toxicity study^a^

		Hemoglobin (g dL^−1^)	RBC count (×10^6^ μL^−1^)	WBC count (×10^3^ μL^−1^)	Platelet count (×10^3^ μL^−1^)
NaCl	(10 mL kg^−1^)	17.46 ± 0.33	4.78 ± 0.12	10.62 ± 1.13	799.34 ± 23.65
ACCE	(300 mg kg^−1^)	14.55 ± 0.34	4.57 ± 0.14	12.18 ± 0.44	812.76 ± 32.90
	(700 mg kg^−1^)	13.82 ± 0.27	5.32 ± 0.15	9.62 ± 0.14	819.54 ± 25.88
GIAE	(300 mg kg^−1^)	14.33 ± 0.55	5.11 ± 0.13	11.54 ± 1.14	811.22 ± 27.66
	(700 mg kg^−1^)	15.72 ± 0.66	5.32 ± 0.10	13.34 ± 0.83	784.78 ± 33.55

a ACCE/GIAE actions reflected by indicated hematological parameters and assessed in healthy and treated rats. Data are expressed as mean ± standard error (*n* = 6).

**Table 3:** Effects of ACCE and GIAE on biochemical parameters in sub-acute toxicity study^a^

		Triglycerides (mg dL^−1^)	Total cholesterol (mg dL^−1^)	Creatinine (mg dL^−1^)	ALT (U L^−1^)	AST (U L^−1^)
NaCl	(10 mL kg^−1^)	72.35 ± 5.17	85.65 ± 5.71	1.52 ± 0.16	45.04 ± 1.19	55.27 ± 2.39
ACCE	(300 mg kg^−1^)	64.12 ± 4.21	87.94 ± 6.12	1.77 ± 0.21	49.15 ± 3.77	58.51 ± 2.44
	(700 mg kg^−1^)	67.11 ± 5.10	91.52 ± 5.16	1.58 ± 0.14	47.66 ± 2.37	60.21 ± 1.28
GIAE	(300 mg kg^−1^)	64.55 ± 3.24	87.47 ± 3.18	1.48 ± 0.10	44.22 ± 3.56	54.17 ± 2.11
	(700 mg kg^−1^)	73.81 ± 4.00	95.44 ± 4.23	1.55 ± 0.23	50.33 ± 3.94	57.91 ± 3.23

a ACCE/GIAE actions reflected by indicated biochemical parameters and assessed in healthy and treated rats. Data are expressed as mean ± standard error (*n* = 6).

### 3.3. Opposite gastric-emptying actions of extracts and drug

**Fig. 3:**
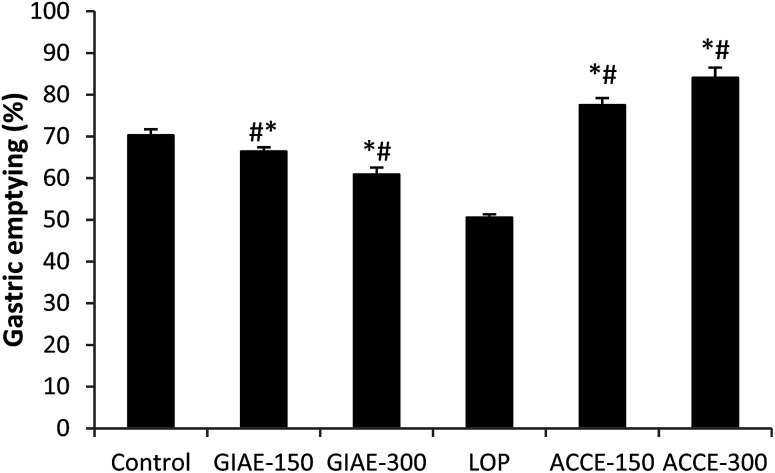
Effect of ACCE/GIAE or LOP on gastric emptying. Animals were treated 1 h prior to a test meal (50 mg of phenol red in 100 mL of aqueous methyl cellulose) with diverse doses of ACCE/GIAE (150 and 300 mg kg^−1^). Results are expressed as mean ± SEM; *n* = 6 in each group. Data was analysed by StatView ANOVA. *p < 0.05 compared to control group, ^#^*p* < 0.05 compared to LOP-group.

In the LOP (5 mg kg^−1^) induced delayed gastric emptying model, ACCE was found to possess a remarkable GE acceleration at orally administered doses of 150 and 300 mg kg^−1^, showing increases of 77% and 84%, respectively, in comparison to healthy rats (70%) and the LOP group (50%). In contrast, GIAE at two doses (150 and 300 mg kg^−1^) exerted a decrease in GE (66% and 60%, respectively) (Fig. 3), which revealed the opposite activity of the two extracts of *Cinnamomum cassia* bark and *Quercus ilex* L.

### 3.4. Reverse effects of extracts and antagonistic drugs on small bowel transit

In this context, charcoal meal was used as a marker in the estimation of GIT. The study showed a significant increase (*p* < 0.05) in GIT in the normal rats treated with 150 mg kg^−1^ and 300 mg kg^−1^ of ACCE. ACCE exerted an effective laxative action, depending on the dose of GIT (24–24%) compared to 30% and −54.53% caused by standard drugs (yohimbine and clonidine) (Table 4). However, in our previous research, different doses of GIAE (125, 250 and 500 mg kg^−1^) administered orally caused potent dose-related inhibition of intestinal meal travel distance in healthy rats. In fact, the highest intestinal transit reduction of 49% was obtained with 500 mg kg^−1^ compared to 58% caused by the reference drug (clonidine, 1 mg kg^−1^).^28^

**Table 4:** Effects of ACCE and reference drugs (clonidine and yohimbine) on gastrointestinal motility in rat^a^

Rat-groups	Total small intestine length (cm)	GI-motility		
		Transit distance of charcoal meal (cm)	GI-charcoal transit (%)	Acceleration/inhibition (%)
Control (10 mL kg^−1^)	83.65 ± 5.66	66.25 ± 3.18	79.20 ± 3.04	—
Clonidine (1 mg kg^−1^)	77.27 ± 4.11	30.12 ± 2.77*	39.00 ± 2.47*	54.53
Yohimbine (2 mg kg^−1^)	86.27 ± 5.02	86.03 ± 4.21*	99.72 ± 2.38*	30.00
Aqueous extract (ACCE) 150 mg kg^−1^	87.40 ± 3.68	75.21 ± 2.56^#^*	86.05 ± 4.34^#^*	14.00
300 mg kg^−1^	86.45 ± 3.17	82.37 ± 2.162^#^*	95.28 ± 3.16^#^*	24.33

a ACCE/drugs modulations reflected by indicated parameters and assessed in healthy and treated rats. Data are expressed as mean ± standard error (*n* = 7); *p < 0.05 *versus* control group, ^#^*p* < 0.05 *versus* yohimbine/clonidine-groups.

### *3.5*. Laxative action of ACCE on (LOP)-drug-induced constipation in rats

To test the laxative action of ACCE treatment on LOP-induced constipated rats, fecal parameters, including stool numbers, wet/dry weights and water contents were determined. As shown in Table 5, stool numbers and weights were significantly decreased after administration with the standard drug, while both doses of ACCE (150 and 300 mg kg^−1^) were shown to increase these fecal parameters compared with those in constipated rats and the healthy group. However, in our previous study, GIAE administration at diverse doses (125, 250 and 500 mg kg^−1^) had a preventative effect against diarrhea in rats.^31^ These results demonstrated that the extract treatments improved two opposite GI disorders (constipation and diarrhea) in rats through the enhancement of fecal parameters.

**Table 5:** Fecal parameters after LOP caused acute-constipation and protective action of ACCE^a^

Rats-group	Fecal parameters on day 5 (collection for 24)			
	Fecal pellet (*n*)	Wet weight (g/24 h/rat)	Dry weight (g/24 h/rat)	Water content (%)
CONT (NaCl)	52.37 ± 2.56	4.91 ± 0.55	2.02 ± 0.22	42.15 ± 3.66
LOP (5 mg kg^−1^)	40.23 ± 1.54*	2.74 ± 0.41*	1.03 ± 0.14*	22.43 ± 1.83*
ACCE (150 mg kg^−1^)	44.53 ± 3.51*	3.23 ± 0.51*	1.55 ± 0.32*	28.24 ± 1.66*
(300 mg kg^−1^)	48.35 ± 3.60^#^	4.32 ± 0.33^#^	1.84 ± 0.01^#^	37.91 ± 4.15^#^

a ACCE/drug moderations reflected by indicated fecal-parameters and assessed in healthy and constipated rats. Data are expressed as mean ± standard error (*n* = 7); *p < 0.05 *versus* control group, ^#^*p* < 0.05 *versus* LOP-group.

### 3.6. ACCE-attenuated LOP-induced altered oxidative stress in constipated rats

To evaluate whether oxidative stress response can play a crucial role in the induction and treatment of LOP-induced constipation in rats, the main parameters of oxidative indicators and antioxidants are studied. The administration of ACCE at two increasing doses leads to a protective effect against all macromolecular alterations (protein carbonylation, lipid oxidation and antioxidant enzyme perturbation) caused by massive ROS production following LOP intoxication and constipation initiation in rats. Indeed, the extract showed an efficacious action and re-established oxidative status in the colon by decreasing the levels of MDA and carbonyl proteins, and improving the thiol groups and action of antioxidant enzymes, especially SOD and CAT (Table 6).

**Table 6:** Oxidative indicators modulation after LOP caused acute-constipation and corrective action of ACCE^a^

		MDA (10^−3^ nmol mg^−1^ proteins)	–SH (nmol mg^−1^ proteins)	Carbonylated-proteins (μmol mg^−1^ proteins)	SOD (10^−3^ U mg^−1^ proteins)	CAT (10^−3^ mol min^−1^ mg^−1^ proteins)
NaCl	(5 mL kg^−1^)	30.45 ± 3.45	77.22 ± 6.45	19.54 ± 1.523	138.44 ± 7.30	296.67 ± 11.11
LOP	(5 mg kg^−1^)	95.23 ± 5.56*	37.00 ± 2.77*	39.29 ± 3.91*	79.18 ± 5.91*	188.48 ± 9.00*
ACCE	(150 mg kg^−1^)	64.34 ± 4.17*^#^	47.24 ± 4.21*^#^	32.43 ± 2.33*^#^	84.11 ± 6.33*	227.81 ± 8.05*^#^
	(300 mg kg^−1^)	29.45 ± 4.22^#^	68.43 ± 4.50*^#^	20.28 ± 0.94^#^	111.56 ± 5.12^#^	279.98 ± 10.77^#^

a ACCE/LOP actions reflected by indicated oxidative stress parameters and assessed in healthy and constipated rats. Data are expressed as mean ± standard error (*n* = 7); *p < 0.05 *versus* control group, ^#^*p* < 0.05 *versus* LOP-group.

### 3.7. Effects of ACCE on LOP-induced intracellular mediator disruption

To investigate cell-level damage, we studied the effect of LOP on intracellular mediators. In this context, we found a significant (*p* < 0.05) increase in H_2_O_2_ production in constipated rats (55.43 ± 3.14 μmol mg^−1^ proteins) compared to the control group (22.56 ± 2.67 μmol mg^−1^ proteins) accompanied by a significant (*p* < 0.05) depletion of calcium and free iron. However, bioactive compounds and mineral constituents that exist in ACCE protect against all these disruptions (Table 7).

**Table 7:** Intracellular-mediators moderation after LOP caused acute-constipation and corrective effect of ACCE^a^

		H_2_O_2_ (μmol mg^−1^ proteins)	Free iron (nmol mg^−1^ proteins)	Calcium (10^−3^ nmol mg^−1^ proteins)
NaCl	(5 mL kg^−1^)	22.56 ± 2.67	24.12 ± 1.32	37.00 ± 2.56
LOP	(5 mg kg^−1^)	55.43 ± 3.14*	14.22 ± 0.78*	19.55 ± 0.95*
ACCE	(150 mg kg^−1^)	34.90 ± 2.00*^#^	17.01 ± 0.44*^#^	22.53 ± 1.13*^#^
	(300 mg kg^−1^)	23.67 ± 0.21^#^	25.52 ± 2.77^#^	35.28 ± 1.94^#^

a ACCE/LOP effects reflected by indicated intracellular mediators and assessed in healthy and constipated rats. Data are expressed as mean ± standard error (*n* = 7); *p < 0.05 *versus* control group, ^#^*p* < 0.05 *versus* LOP-group.

## 4. Discussion

In the present study, we sought to compare the bioactive components and potential GI-physiological actions of extracts prepared from cinnamon bark and evergreen oak acorn, as well as the possible underlying physiological mechanisms of the therapeutic effects in laboratory animals.

Previous research revealed that cinnamon contained ash (2.4%), crude protein (3.5%), crude fat (4%), crude fiber (33.0%), moisture (5.1%), carbohydrates (52.0%) and polyphenolic compounds especially flavonoids.^32–34^ However, a quantitative analysis of the phytochemical substances of GIAE revealed the abundance in particular of tannins but low amount in fibers and carbohydrates.^31^ Added to that, the comparative phytochemical investigation by HPLC-PDA/ESI-MS showed that the main phenolic compounds of ACCE are *trans*-cinnamic acid, quinic acid, protocatechuic acid, rosmarinic acid, epicatechin and *p*-coumaric acid, while gallic acid, quinic acid, protocatechuic acid, *p*-coumaric acid, syringic acid and naringin constitute the predominant chemical components in GIAE.

Toxicological examinations have revealed that in both acute and sub-acute toxicity tests, ACCE/GIAE did not produce any toxic effect in mice. In the acute toxicity study, no morbidity or mortality were observed in any mice, which survived throughout the 24 h of observation. The LD_50_ value of the extract was found to be greater than 3000 mg kg^−1^. These findings, therefore, suggest that the extracts at the dose limit tested are essentially non-toxic and safe after oral administration. However, previous findings revealed the cytotoxic and genotoxic potential of *Cinnamomum cassia* bark water extract *in vitro*, particularly in high doses or with long-term use of the extract, and this therefore needs to be clarified by *in vivo* studies.^35^

Gastric emptying is the main determinant of nutrient delivery to the small intestine. For example, an upper gastrointestinal motor function, particularly the gastric-emptying rate, is a major determinant of levels of postprandial blood glucose, and there is developing support for the concept that gastric-emptying modulation could be used to optimize glucose control in diabetes.^36^ On the other hand, GI-transit time may be also an important determinant of glucose homeostasis and metabolic health through actions on the intestinal-absorption process of nutrients and microbial composition, among other mechanisms. Thus, an alteration in GI transit may play a crucial role in the etiology of metabolic diseases such as type 2 diabetes mellitus and obesity (T2DM).^37^ Consequently, GI-function moderation may be one of the mechanisms underlying beneficial health effects, by regulating these disturbances with dietary fibers and other physiologically functional components. For this reason, the separate administration of extracts in rats showed opposite effects on gastric emptying and GIT. These actions are strongly due to the unequal distribution or presence/absence of physiologically bioactive compounds. Indeed, soluble dietary fibers and carbohydrates promoted beneficial physiological gastrointestinal effects, such as laxative activity.^21^ Soluble fibers have frequently been reported to stimulate/accelerate peristaltic activity by maintaining higher acidity levels because fiber fermentation results in SCFA production by intestinal coliform bacteria in rats.^38^

*trans*-Cinnamic acid (TrCin), an organic acid, and its derivatives were investigated for modulating the activity of gastric emptying. The GE experiment was conducted to confirm improved delayed gastric-emptying. Therefore, six of the cinnamic derivatives tested, that is cinnamic acid, *p*-methoxycinnamic acid, 3,4,5-trimethoxycinnamic acid, 2-(trifluoromethyl) cinnamic acid, 3-(trifluoromethyl) cinnamic acid, and trans 4-(trifluoromethyl) cinnamic acid, ameliorated gastric emptying delay compared with the control.^39^ Furthermore, the structure–activity relationships suggest that rosmarinic acid, *p*-coumaric acid and *trans*-cinnamic acid, and its derivatives should be evaluated for their α-glucosidase inhibitory action.^40–42^ The inhibition of α-glucosidase extends gastric emptying and leads to satiety and weight loss.^43^ However, tannins affect intake by slowing the action of digestion and emptying of the digestive tract, and stimulating the nervous system to inhibit further intake of food. Loss of palatability could be a result of reactions between tannins and salivary muco-proteins, or a direct reaction with taste receptors, provoking an astringent sensation. Tannins denature proteins in the intestinal mucosa by forming protein tannates, which make the intestinal mucosa more resistant to chemical alteration and reduce secretion.^44^

Added to that, a remarkable decrease in fecal excretion in LOP-induced constipated rats is considered one of the key markers of constipation in several constipation studies. Previously, stool-related factors, such as stool number, weight, and water content were shown to have appreciably declined in rats upon administration of LOP.^22^ However, these alterations were significantly recovered by ACCE containing physiologically bioactive components with laxative action, by improving the number, water content, and weight of stools in LOP-induced constipation in a dose-dependent manner. Functional food including Zhizhuwan, fat-free milk, *Dendrobium officiate*, *Lactobacillus fermentum*, and naringenin are known to relieve the symptoms of chronic constipation.^45^

Many recent studies have shown the contribution of stress to the development of certain gastrointestinal disorders, including ulcers, constipation, diarrhea and colitis. Indeed, excessive production of ROS as H_2_O_2_ is accompanied by an alteration in macromolecules such as proteins, lipids, and DNA, which causes the dysfunction of certain digestive enzymes, enterocyte membranes and gene expression. These alterations induced, in turn, the disruption of the intestinal absorption/secretion process of water and electrolytes.^21^ Our results also demonstrate that the laxative effect of ACCE is associated with the functional recovery of gastrointestinal oxidative stress in the LOP-induced constipation model. It has been shown that the major antioxidant constituents are phenolic compounds. The positions of the active groups play an important role in structure–antioxidant relationship activity. Their antioxidant activity seems to be related to their molecular structure, or more precisely to the presence and number of hydroxyl groups, and to double bond conjugation and resonance properties.^46^ Moreover, these results provide additional evidence that gastrointestinal antioxidant capacity can be considered a key marker to evaluate the laxative effects of natural products. An alternative hypothesis is that antioxidant supplements modulate endogenous mechanisms that either decrease ROS production or increase the enzyme activities that decompose ROS.^47^ In this context, Li *et al.*^48^ showed that pre-treatment with cinnamic acid might result in a certain reduction in lipid peroxidation and prompt tolerance to chilling stress by means of boosting the activities of antioxidants such as SOD, CAT, GPX and GSH. Also, protocatechuic acid was shown to have strong free radical scavenging and antioxidant activities by decreasing lipid peroxidation, diminishing oxidized low-density lipoprotein levels (LDL), reducing the production of hydrogen peroxide (H_2_O_2_) and superoxide anions and also by restoring glutathione (GSH)-related enzymes.^49^

Regulation of gastrointestinal hormones, such as cholecystokinin (CCK), gastrin (GAS), somatostatin (SS) and motilin (MTL), may also be another important mechanism for improving the symptoms of LOP-induced constipation in animal models after ACCE administration. The moderation of these substances by herbal products has been examined as one of the strategies for treating functional constipation.^50^ Moreover, various studies have revealed disruptions in the composition and stability of the intestinal microbiota in constipated subjects compared with healthy controls. These perturbations may influence gastrointestinal motility and secretory actions by changing the amount of available physiologically active substances and the metabolic environment of the gut. Recent studies have documented that dietary polyphenols contribute to the upkeep of intestinal health and motility, by maintaining the gut microbial balance through the stimulation of the production of beneficial bacteria and the inhibition of pathogenic bacteria.^51^

It has been mentioned in several studies that the interstitial cells of Cajal (ICCs) contribute to normal GI function by generating electrical slow waves and mediating neuromuscular signaling. Damage to ICCs or diminution in ICC numbers have been described in many GI motility disorders, such as constipation.^52^ For this reason, we believe that ACCE can depolarize the membrane potentials of these cells. This excitation may be led to smooth muscle cells *via* the gap junction. ICCs might respond to this depolarization with activation of the voltage-dependent calcium channels.^53^ Therefore, this effect of pacemaker potential depolarization might induce an intestinal-motility augmentation, as with various others medicinal plants including *Liriope platyphylla*^54^ and *Citrus unshiu*.^55^

Another possible mechanism could be the smooth muscle contractions which are known to depend on the intracellular calcium level. Because natural compounds caused an stimulation/inhibition of the contractions of the ileum, which involved an increase/decrease in cytosolic calcium, either by stimulating/inhibiting calcium influx or by releasing/inhibiting calcium liberation from intracellular stores, or both. The exciting/relaxant actions could also have resulted from other mechanisms, such as an increase/decrease in the sensitivity of the contractile apparatus to existing concentrations of calcium and/or stimulation/inhibition of the binding of calcium to the contractile proteins.^56^ Further studies are required to evaluate the exact mechanism of action of GIAE/ACCE on gastrointestinal smooth muscles and functions/disorders.

## 5. Conclusion

These findings lead us to conclude that aqueous extracts of both *Cinnamomum cassia* bark and *Quercus ilex* L. contain diverse/different functional ingredients with opposite actions on gastrointestinal-physiological and unbalanced GI functions, such as diarrhea and constipation. These findings may help future studies to derive the underlying mechanisms and the potential molecular targets of GIAE/ACCE bioactive substances for structure–relationship activities and the development of physiologically functional foods and future therapies.

## Financial disclosures

None declared.

## Conflicts of interest

Only the authors are responsible for the content of this paper.

## Abbreviations

ACCE: *Cinnamomum cassia* bark aqueous extract
CCK: Cholecystokinin
CON: Constipation
DGE: Delayed gastric emptying
FGID: Functional gastrointestinal disorders
GAS: Gastrin
GE: Gastric emptying
GET: Gastric emptying time
GIAE: *Quercus ilex* aqueous extracts
GIT: Gastrointestinal transit
HPLC-PDA/ESI-MS: Liquid chromatography-photodiode-array-mass spectrometry
ICCs: Interstitial cells of Cajal
IBS: Irritable bowel syndrome
LOP: Loperamide
MTL: Motilin
SS: Somatostatin
TrCin: *trans*-Cinnamic acid

## Supplementary Material
